# Cloning and transcriptional expression of a novel gene during sex inversion of the rice field eel (*Monopterus albus*)

**DOI:** 10.1186/s40064-015-1544-z

**Published:** 2015-12-01

**Authors:** X. C. Qu, J. Y. Jiang, C. Cheng, L. Feng, Q. G. Liu

**Affiliations:** College of Fisheries and Life Sciences, Shanghai Ocean University, Shanghai, 201306 China; College of Life Sciences, Guangxi Normal University, Guilin, 541004 China; Key Laboratory of Ecology of Rare and Endangered Species and Environmental Protection, Ministry of Education, Guangxi Normal University, Guilin, 541004 China

**Keywords:** Differentially expressed genes (DEGs), Ovotestis, DDRT-PCR, Hermaphroditic fish

## Abstract

We performed annealing control primer (ACP)-based differential-display reverse transcription-polymerase chain reaction (DDRT-PCR) to isolate differentially expressed genes (DEGs) from the stage IV ovary and ovotestis of the rice field eel, *Monopterus albus*. Using 20 arbitrary ACP primers, 14 DEG expressed-sequence tags were identified and sequenced. The transcriptional expression of one DEG, *G2*, was significantly greater in the ovotestis than the stage IV ovary. To understand the role of *G2* in sex inversion, *G2* cDNA was cloned and semi-RT-PCR, real time PCR were performed during gonad development. The full-length *G2* cDNA was 650 base pairs (bp) and it comprised a 5′-untranslated region (UTR) of 82 bp, a 3′-UTR of 121 bp and an open reading frame of 444 bp that encoded a 148-amino acid protein. The expression of *G2* was weak during early ovarian development until the stage IV ovary, but expression increased significantly with gonad development. We speculate that *G2* may play an important function during sex inversion and testis development in the rice field eel, but the full details of the function of this gene requires further research.

## Background

In vertebrates, sex can be determined by one of many mechanisms, including chromosomal, polygenic and environmental sex determination. There are approximately 24,000 species of fish (Nelson 1994), and examples of each mechanism exist within fish species. Indeed, in some fish, more than one mechanism can contribute to sex determination and different species exhibit various mechanisms for controlling sexual determination and patterns of sexual differentiation. The study of gender control not only has important theoretical applications, but a greater understanding of these mechanisms also has considerable practical utility. Sex determination and differentiation in fish is a very promiscuous process with respect to evolutionary patterns observed between genera and families, and within individuals subjected to modifications by external factors (Devlin and Nagahama 2002; Herpin and Schart 2011). Sex determination is the genetic and environmental processes, whereas sex differentiation is the physical realization of genetic variability and environmental factors in terms of testicular or ovarian development (Baroillier et al. 1999; Nakamura et al. 1998; Uller and Helantera 2011). In contrast to the well-established studies on sex determination in humans and a few animals (Sinclair et al. 1990; Matsuda et al. 2002; Yoshimoto and Ito 2011; Angelopoulou et al. 2012), although a series of studies have been conducted in hermaphrodite fish over recent years (Alam et al. 2008; Zhou and Gui 2010; Nozu et al. 2009; Wu et al. 2010; Jeong et al. 2009), no breakthroughs in progress have been made to date and the molecular mechanisms that govern sex determination and differentiation in many hermaphrodite fish remain elusive. Furthermore, the genetic mechanisms of sex determination (including sex inversion) are known only for some gonochoristic fish species (Wu et al. 2012).

The rice field eel (*Monopterus albus*) is an economically important freshwater fish distributed widely in China, Japan and other parts of Southeast Asia (Xiao and Liu 1995a). It is a protogynous freshwater fish that exhibits an adult sex inversion from functional females to males (Liu 1944; Liem 1963; Chan and Phillips 1967; Xiao and Liu 1995b). According to Xiao and Liu (Xiao and Liu 1995a, b), the female sexual development of *M. albus* can be divided into six stages, termed I to VI. After sex maturity and ovulation is achieved, the ova and ovarian gradually degenerate. At the same time, spermatogonia begin to multiple on the germinal fold and form spermatocysts. This is the intersex development stage, and the male individual then develops from the female. The male development of *M. albus* can also be divided into different stages. This eel is commonly studied because of these unique physical characteristics.

In recent years, environmental pollution and habitat degradation in China have dramatically reduced the populations of rice field eel. Enhanced understanding of the biological mechanisms of sex inversion should help relieve the present urgent market requirements for rice field eels seedlings. In most cases, the reproductive capacity of these species is compromised, and hence the study of strategies and mechanisms involved in reproduction and sexual differentiation are central to evaluate the potential effects of contaminants. Recently, great efforts have been made to uncover the mechanism that underlies gonad sex inversion in the rice field eel, including physiological (Chu 2011; Yuan et al. 2012) and molecular aspects (Ye et al. 2007; Xiao et al. 2010; He et al. 2010, 2012; Qu et al. 2011; Huang et al. 2005). However, the mechanism of sex inversion in the rice field eels remains unclear.

Molecular biology techniques have been developed that can identify DEGs in cells under various physiological stages or experimental conditions. Difficulty often arises in identifying the gene responsible for a specialized function during a certain biological stage because the gene is expressed at low levels, whereas the bulk of mRNA within a cell is made up of highly abundant transcripts (Wan et al. 1996). Thus, to screen for low concentration DEG transcripts, PCR amplification is required. One screening method, differential-display, requires PCR using short arbitrary primers (Liang and Pardee 1992, 1995). Although this method is simple, rapid and only requires small amounts of total RNA, many investigators have experienced particularly high false-positive rates (Debouck 1995; Wan et al. 1996) and poor reproducibility of their results because of non-specific annealing of the short arbitrary primers. Recently, a new ACP DDRT-PCR method has been developed, and this technology uses a specific primer type to increase both the specificity and sensitivity of the assay (Hwang et al. 2003; Kim et al. 2004).

In this present study, annealing control primer (ACP)**-**based differential-display reverse transcription-polymerase chain reaction (DDRT-PCR), rapid amplification of cDNA ends (RACE), semi-quantitative RT-PCR, real time PCR methods were performed on ovary, ovotestis and testis stages of the rice field eel gonad in an attempt to screen for genes related to rice field eel sex differentiation and understand sex inversion. This study will provide a reference for future studies into sex differentiation of the rice field eel and other teleosts.

## Methods

### Sample collection and histological observations of the gonad

The rice field eels were captured in Chong Ming Island (31°27′–31°5 l′N, 121°09′′–121°54′E), Shanghai, China, in September 2009. All experiments were performed in accordance with the Animal Care and Use Committee guidelines of the Shanghai Ocean University. The abdomen was slit, a small segment of the gonad was freed from the fat and mesenteric tissues, and then this was removed for histological assessment of the sexual status by routine paraffin sectioning according to the structure of the gonads. Samples for histological analysis were kept in Bouin’s solution overnight before being embedded in paraffin. Cross-section (5 μm) were processed and stained with hematoxylin and eosin. The remainder of the gonad sample was frozen immediately in liquid nitrogen and stored at −80 °C for subsequent molecular analyses. At the same time, referring to the method of Zhu et al. (2010), 50–100 mg sections of gonad were fixed overnight at 4 °C in freshly prepared 4 % paraformaldehyde in 100 mM phosphate buffered saline (PBS; pH 7.4). After washing three times with PBS at room temperature, the samples were stored in methanol at −20 °C.

### RNA isolation and cDNA synthesis for the ACP system

Tissues from the stage IV ovary and ovotestis of rice field eels were dissected and snap frozen in liquid nitrogen. Total RNA was isolated (TaKaRa Mini BEST Universal RNA Extraction Kit) and then processed with deoxyribonuclease I (TaKaRa, Dalian). The total RNA was used for synthesis of the first-strand cDNA by reverse transcriptase. Reverse transcription was performed for 1.5 h at 42 °C in a final reaction volume of 20 μL containing 3 μg of purified total RNA, 4 μL of 5× RT buffer, 5 μL of 10 mM dNTPs, 1 μL of 10 μM cDNA synthesis primer dT-ACP1((5′-CTGTGAATGCTGCGACTACGATXXXXX(T)18-3′)) and 1 μL of M-MLV transcriptase (TaKaRa). These mixtures were heated to 94 °C for 2 min to inactivate the reaction, and the first strand cDNA samples were diluted by adding 80 μL of ddH_2_O.

PCR was conducted with 20 pairs of arbitrary ACPs (Table 1) to synthesize the second strand cDNA under annealing conditions (XiangSC and Nam 2005). For the PCR reaction, ovary and ovotestis cDNA samples were used as a template for amplification using different sets of arbitrary ACPs according to the GeneFishing™ Kit (Neuro-Hemin Biotech, Hangzhou). PCR was performed in 25 μL volumes containing 1 μL of cDNA, 2.5 μL of 10× PCR buffer, 1 μL arbitrary ACP and dT-ACP, 1.5 μL of 10 mM dNTP and 0.5 μL Taq polymerase (TaKaRa). After incubating at 94 °C for 2 min, the PCR was performed at 50 °C for 5 min then 72 °C for 1 min, followed by 40 cycles of 94 °C for 30 s, 65 °C for 40 s and 72 °C for 1 min, and finally 72 °C for 7 min. PCR products were electrophoresed on 2 % agarose gels and stained with ethidium bromide.

**Table 1:**
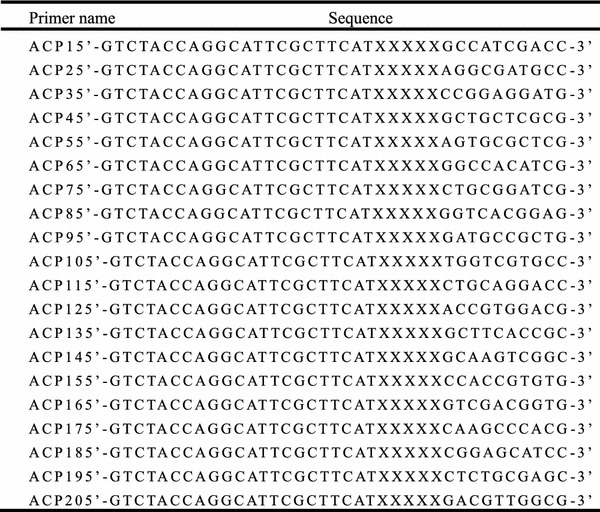
The ACP primer sequences used in this study

### Cloning and transformation

Differentially expressed bands were cloned into the pMD-T(18) vector (TaKaRa). In order to verify the identity of the inserted DNA, isolated plasmids were sequenced using an automated DNA sequencer by Jie Li Biology Co., Ltd. (Shanghai, China). Complete sequences were analyzed by searching for homologous genes using the BLASTn and BLASTx algorithms in the GenBank database (http://www.ncbi.nlm.nih.gov).

### Semi-quantitative RT-PCR and real time PCR

We identified a differentially expressed gene (DEG) called *G2* that had greater levels of transcription in the ovotestis than the stage IV ovary, and this gene was selected to validate the ACP DDRT-PCR prescreening approach. Semi-quantitative RT-PCR and real-time PCR were performed using total RNA from the gonad (ovary at stages II, III, IV, V, VI, the ovotestis, and early and late stage testis) and other tissues (brain, heart, spleen, liver, kidney and muscle) in individual. The early stage testis contains spermatogonia and spermatocytes, while the late stage is defined once the spermatocytes have undergone meiosis and the spermatogenic cysts contain sperm or have released the sperm. First-strand cDNA was synthesized from 2 µg of total RNA from each individual using oligo dT (18) primers (TaKaRa) and the M-MLV reverse transcriptase enzyme (TaKaRa). The RNA transcription levels of G2 were analyzed by semi-quantitative RT-PCR and real time PCR. β-actin was used as the internal control and muscle was the control tissue. The real-time PCR (ABI 7500) cycling conditions were as follows: 3 min at 95 °C, 40 cycles of 15 s at 95 °C, 30 s at 60 °C, and 1 min at 72 °C in a 20-μl reaction mix containing 2 × SYBR Green I (10 μL), primers (10 μM), 50 × Rox Reference Dye II 0.4 μL, cDNA Template 1 μL (20 ng) and ddH_2_O. The primer sequences were 5′-ACGGCAAG.

GTTGTGATGG-3′ (G2 F); 5′-GTTATGGAAGTTGGCAGGGT-3′ (G2 R); 5′-ATCGCCGC ACTCGTTGTTGAC-3′ (β-actin F); and 5′-CCTGTTGGCTTTGGGGTTC-3′ (β-actin R).

### G2 full-length cDNA amplification and sequencing

Full-length *G2* cDNA was retrieved by performing 5′ RACE using the SMART RACE cDNA Amplification Kit and the Advantage 2 PCR Kit (Clontech). Gene-specific primers were designed according to the known expressed-sequence tag (EST). The primer sequence was 5′-GCCACACTTGGAACCCACAACCACAGAGA-3′. Thermal cycle parameters were 25 cycles of 94 °C for 30 s, 68 °C for 30 s, and then 72 °C for 3 min. The RACE products were cloned and sequenced as described above.

### Statistical analysis

All values of real time PCR were processed by SPSS 17.0 software, and calculated as the 2^−ΔΔCt^ mean ± standard deviation. Differences between two consecutive stages of gonad development were tested using DunnettT3 software. Differences were considered significant if P was ≤0.05 and highly significant if P was ≤0.01. ΔΔCt was calculated as follows: (Ct_target_–Ct_actin_)_the gonad stage_ −(Ct_target_−Ct_actin_)_the stage II ovary_ (Qu et al. 2014).

## Results

### Gonad histology

**Fig. 1:**
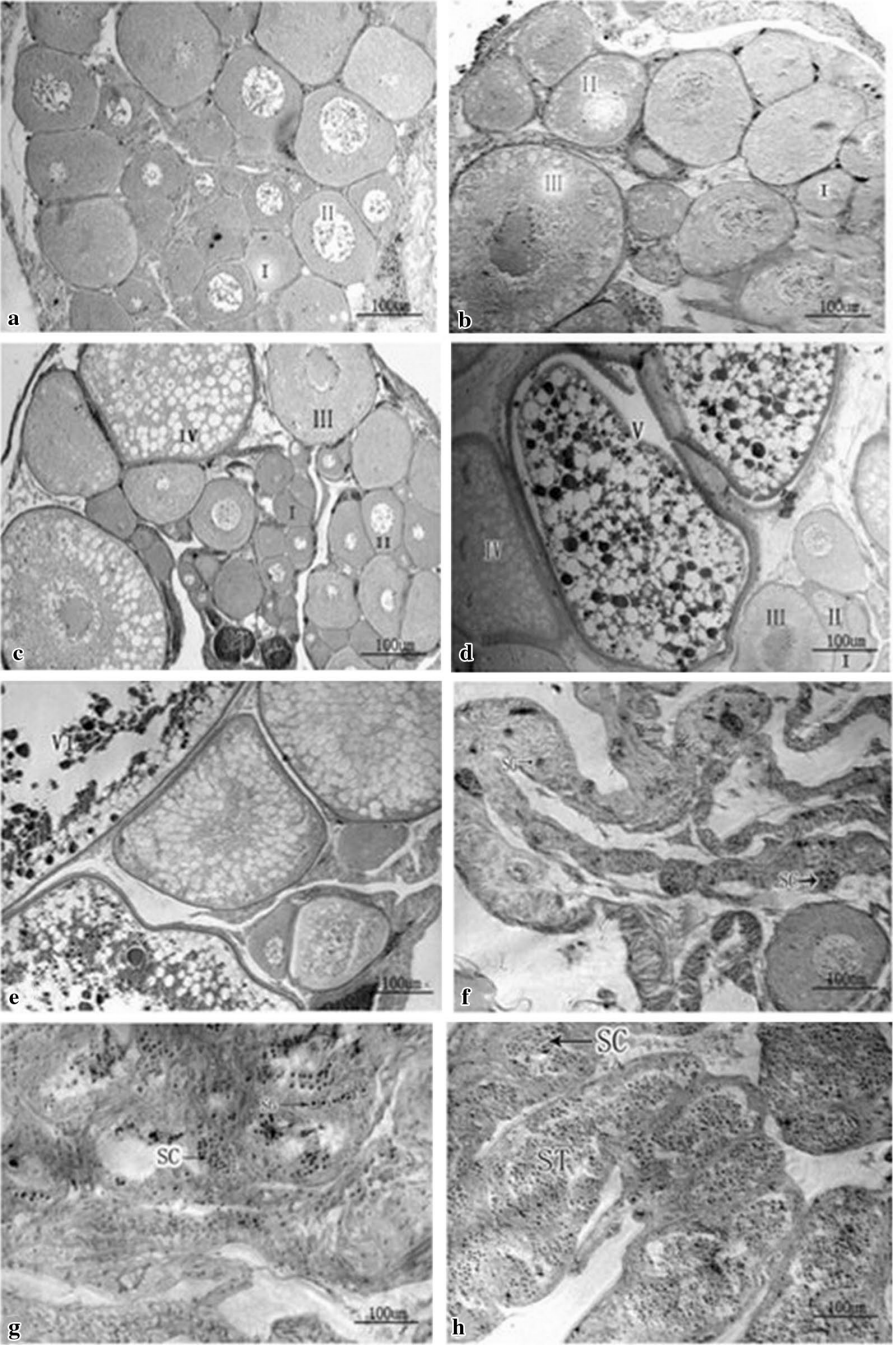
Histological sections from the gonads of the rice field eel (*Monopterus albus*). Ovarian of stage II (**a**), ovarian of stage III (**b**), ovarian of stage IV (**c**), ovarian of stage V (**d**), ovarian of stage VI (**e**), ovotestis (**f**), early testis (**g**) and latter testis (**h**). I: ovary follicle of stage I; II: ovary follicle of stage II; III: ovary follicle of stage III; IV: ovary follicle of stage IV; V: ovary follicle of stage V; VI: ovary follicle of stage VI; *SG* spermatogonia, *SC* spermatocyte, *ST* spermatid

We harvested eight gonad stages from rice field eels during sex development: ovaries in stages II, III, IV, V, and VI, the ovotestis, and early and late testis (Fig. 1). Because the paraffin sections showed there were no male tissues in the stage IV ovary tissues while the spermatocytes first appeared in ovotestis, we decided to isolate DEGs from the gonad tissues at these stages.

### Identification of DEGs between the stage IV ovary and ovotestis tissues

**Fig. 2:**
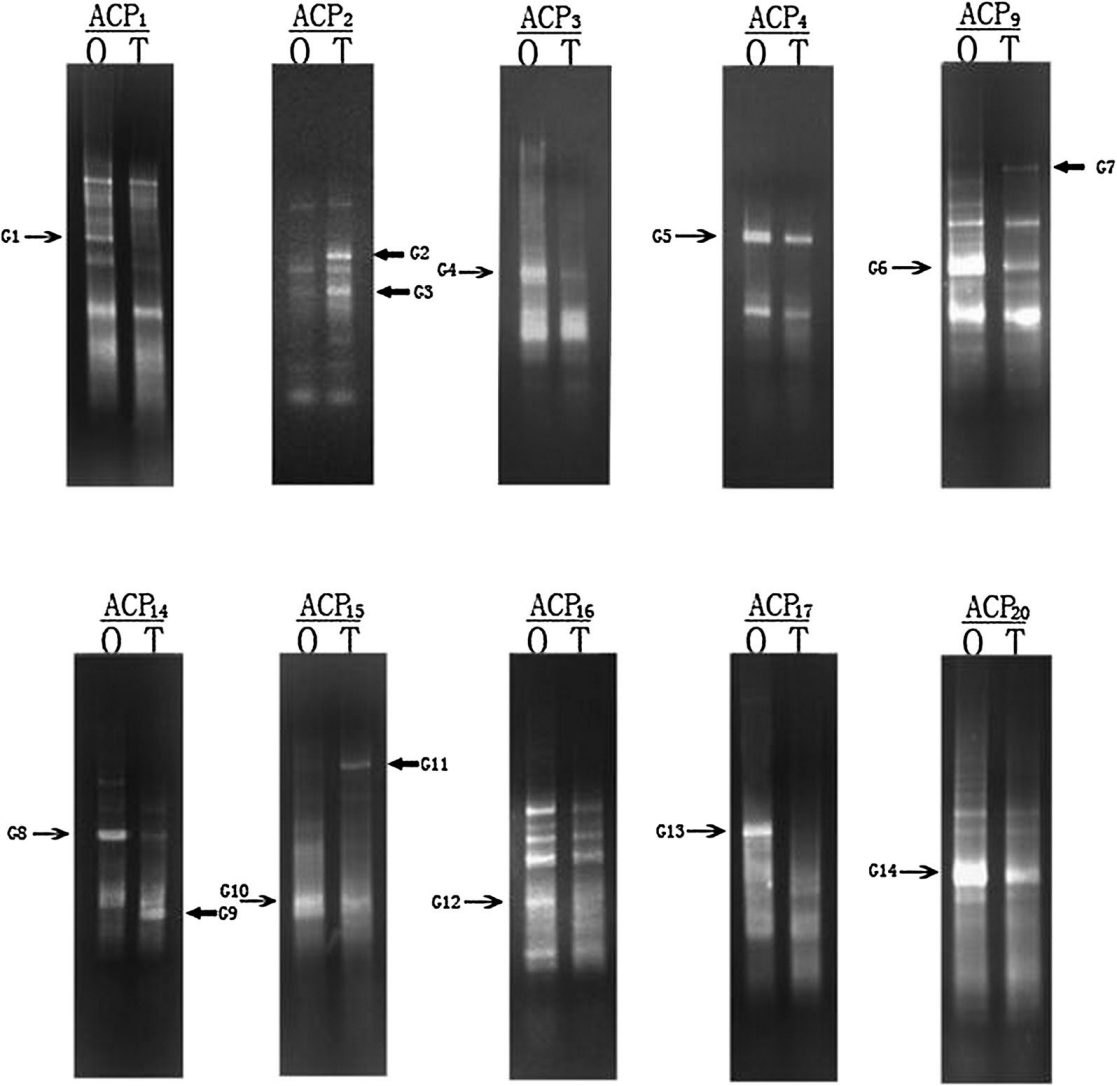
The results of ACP DDRT-PCR obtained from the stage IV ovary (O) and ovotestis (T). *O* stage IV ovary, *T* ovotestis. The ACP DDRT-PCR products were separated on 2 % agarose gel and stained with ethidium bromide. ACP1-20 indicates the arbitrary primers that were used in the ACP DDRT-PCR technique. The *arrow* indicates the differentially expressed genes (G1-14) identified between the stage IV ovary and the ovotestis

We performed ACP DDRT-PCR to identify DEGs between the stage IV ovary and ovotestis tissues using a combination of 20 arbitrary primers and two anchored oligod T_(18)_ primers from an ACP-based GeneFishing PCR kit (Seegen Inc.). A total of 14 DEG fragments were isolated from gels and sequenced. We identified nine down-regulated candidate genes and five up-regulated candidate genes during the sex inversion stage of the rice field eel (Fig. 2). Gene ontology annotations, BLASTN and BLASTX searches of all 14 sequences against the GenBank database revealed that nine genes had strong homology with known genes, whereas five were hypothetical genes (Table 2).

**Table 2:** Sequence homology and characterization of differentially expressed genes

Clone no.	Fragment size (bp)	GenBank homology no.	Homology	Expression pattern	Homologous gene
G1	555	AB212971.1	370/430 (86 %)	Down	Pagrus major imp alpha mRNA
G2	408	–	–	Up	
G3	211	–	–	Up	
G4	421	GO657149.1	246/292 (84 %)	Down	Yellow perch ovarian library 3, mRNA
G5	745	GR673969.1	338/405 (83 %)	Down	Tilapia adult ovary library Oreochromis niloticus cDNA 5,mRNA
G6	479	FD698341.1	351/490 (71 %)	Down	Hippoglossus hippoglossus cDNA, mRNA
G7	785	FE214073.1	212/273 (77 %)	Up	Dissostichus mawsoni adult liver library, mRNA
G8	625	AY614597.1	220/292 (75 %)	Down	Oncorhynchus mykiss myc-regulated DEAD box protein mRNA
G9	211	–	–	Up	
G10	207	BX927294.7	79/91 (86 %)	Down	Zebrafish DNA sequence from clone CH211-222I16 in linkage group 13
G11	939	–	–	Up	
G12	266	–	–	Down	
G13	665	EX465831.1	466/521 (89 %)	Down	Lates calcarifer cDNA clone LBE39E07 5-, mRNA
G14	467	EB506952.1	119/142 (83 %)	Down	Striped sea bream hepatic library Lithognathus mormyrus cDNA, mRNA

*Up* up-regulated candidate genes during sex inversion, *down* down-regulated candidate genes during sex inversion

### Semi-quantitative RT-PCR and real time PCR of G2

**Fig. 3:**
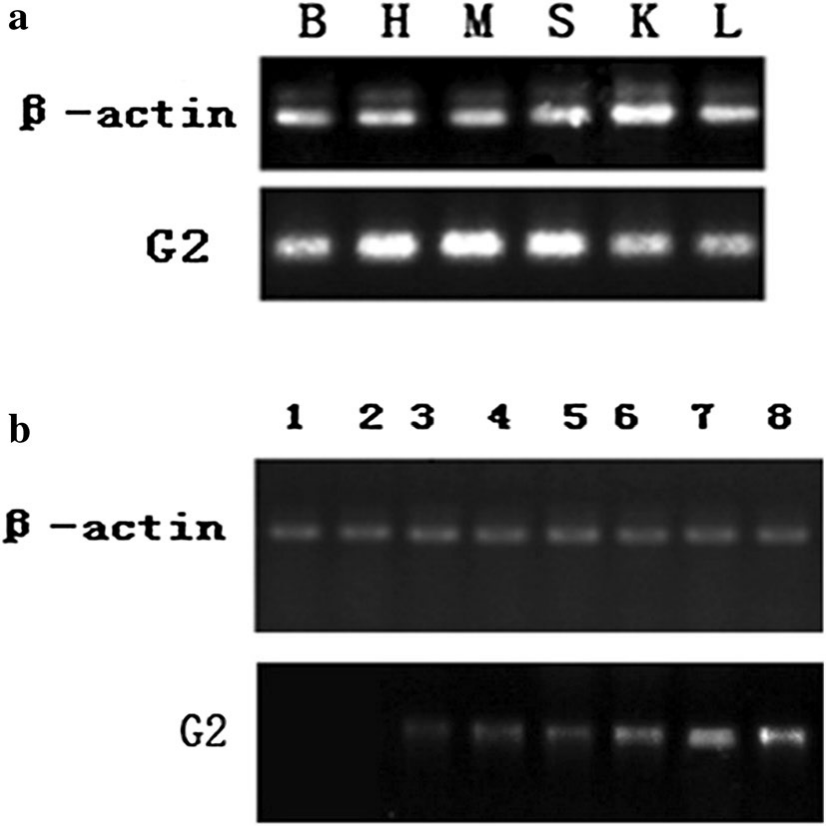
**a** RT-PCR analysis of *G2* transcription in various tissues and gonad stages. *B* brain, *H* heart, *M* muscle, *S* spleen, *K* kidney, *L* liver. **b** RT-PCR analysis of *G2* transcription in various gonad stages. *β*-*actin* was used as the positive control. 1: stage II ovary; 2: stage III ovary; 3: stage IV ovary; 4: stage V ovary; 5: stage VI ovary; 6: ovotestis; 7: early stage of testis; 8: late stage of testis

**Fig. 4:**
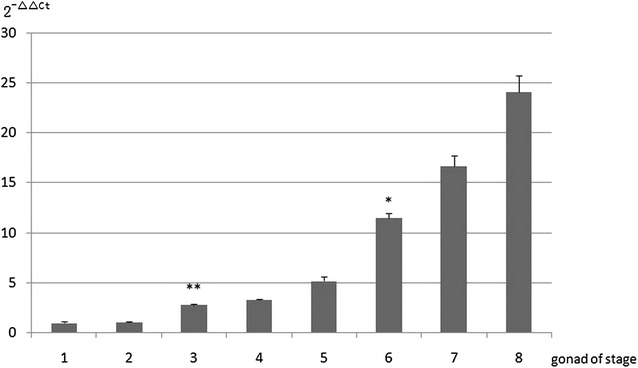
Real time RT-PCR analysis of *G2* transcription expression during various gonadal stages. 1: stage II ovary; 2: stage III ovary; 3: stage IV ovary; 4: stage V ovary; 5: stage VI ovary; 6: ovotestis; 7: early stage of testis development; 8: late stage of testis development. *Asterisk* and *double asterisk symbol* indicate significantly different compared to the former group (*P < 0.05 and *P < 0.01, respectively)

To further determine the specific transcription patterns of one DEG, *G2* expression was analyzed by semi-quantitative RT-PCR and real time PCR. *G2* was expressed in the brain, heart, muscle, spleen, kidney and liver (Fig. 3). On the other hand, semi-quantitative RT-PCR and real time PCR results showed that *G2* was expressed at only low levels in the early developmental stages of the ovary (stages II and III), but it increased significantly and rapidly during gonad development in ovary stages IV, V and VI, the ovotestis, and early and late stages of testicular development (Figs. 3 and 4).

### Cloning and sequence analysis of the full-length G2 gene cDNA

**Fig. 5:**
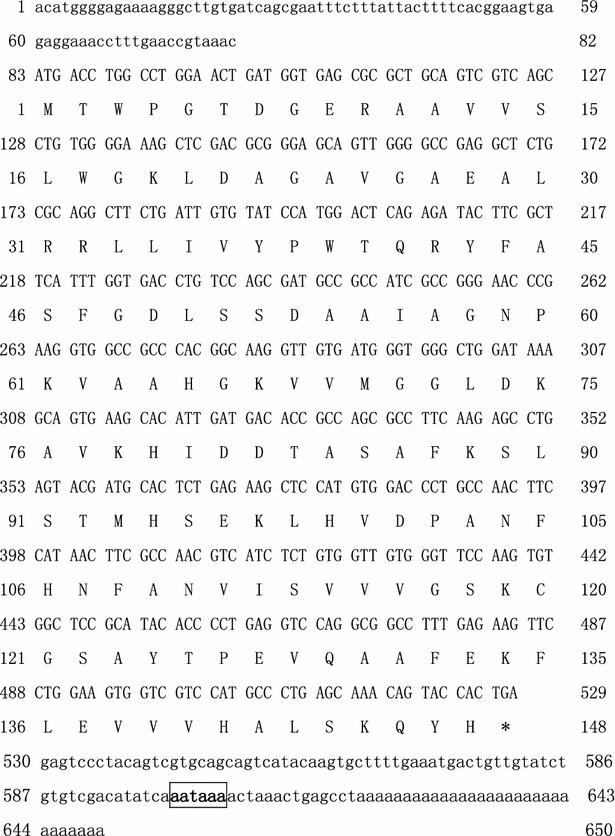
Nucleotide sequence and amino acid sequence of G2.The 5′ and 3′ untranslated regions are in *lower cases*; the polyadenylation signal (AATAAA) is *bold* and *boxed*

The RACE method was used to clone the full-length cDNA of *G2* (650 bp), and this was found to comprise a 5′-untranslated region (UTR) of 82 bp, a 121-bp 3′-UTR and an open reading frame of 444 bp that encoded a 148-amino acid protein (Fig. 5). Similarity analysis showed that *G2* was a hypothetical gene with no homology to any annotated DNA sequences. It is predicted to be a non-classical secretion protein rather than a signal peptide according to SignalP online software (http://www.cbs.dtu.dk/services/SignalP/).

## Discussion

To date, attempts to screen DEGs in the rice field eel have been sparse. The identification of novel gonad specific genes to understand the sex reversal of the rice field eel is therefore of great interest. In this study, in order to characterize the transcriptome in *Monopterus albus*, we used the newly developed ACP DDRT-PCR method and identified 14 DEGs between the ovary and ovotestis that might be involved in gonad development or sex inversion in the rice field eel. Nine genes showed strong levels of homology with known genes, whereas the others were hypothetical genes. Based on our results, we propose that the ACP-DDRT-PCR technique is suitable for the screening of DEGs.

Of the five hypothetical genes, we found that the transcription of *G2* was much higher in the ovotestis than the ovary. Therefore, we cloned *G2* and performed semi-quantitative RT-PCR, real time PCR and in situ hybridization to analyze transcription patterns. Signal peptide prediction showed that *G2* is a non-classical secreted protein gene. According to previous reports (Zhang et al. 2009), most non-classical secreted proteins have high levels of biological activity. Semi-quantitative RT-PCR and real time PCR analysis showed that *G2* transcriptional expression was low in the stage II and III ovaries, but expression was higher in the stage IV ovary and during the later stages of gonad development. We obtained the converse results with regards to the expression profiling of the *JUK1* gene (Xiao et al. 2010), The c-Jun N-terminal kinases (JNKs) are members of the mitogen-activated protein kinase family. Their functions in regulating animal development have been well studied in both invertebrates and vertebrates. On the basis of Xiao’s research, JNK1 plays an important role in sexual reversal of the rice field eel. In a previous paper (Liu 2009), a similar expression pattern was found during sex inversion of the rice field eel, whereby P450arom was predominantly expressed in the ovary, much less in the ovotestis, and barely in the testis. Conversely, P45011β was markedly up-regulated at the onset of testicular development. Steroids play an important role in the sex reversal of hermaphroditic teleosts. More research needs to assess whether G2 is related to steroid secretion or the regulation of steroidogenic genes. In addition, *G2* transcription was detected in the brain, heart, muscle, spleen, kidney and liver. Several papers have reported the gene that have this type of expression profile may be involved in gonadal differentiation (Yu and Tang 2008; Wang et al. 2003). Sex differentiation and development are complex, the regulation of sexual differentiation and sex change processes involves coordinated interactions between genetic, hormonal and environmental factors (Devlin and Nagahama 2002). The number of genes known to be involved in sexual differentiation and inversion in teleosts is increasing (Wu et al. 2010). The purpose of our study was to isolate a sex-determining gene, and the current findings shed light on sex differentiation and sex change in the rice field eel.

## Conclusion

We used ACP DDRT-PCR to analyze DEGs between the stage IV ovary and ovotestis of the rice field eel. We identified 14 DEGs that could be involved in gonad development or sex inversion. Of these, nine genes have strong homology with known genes, whereas the others are hypothetical genes. One of these hypothetical genes, *G2*, showed higher transcription in the ovotestis than in the ovary. We cloned *G2* and used semi-quantitative RT-PCR, real time PCR to analyze its expression. *G2* may play a role in the sex inversion process or testis development in the rice field eel.
